# Physicians and nurses’ knowledge and attitudes towards advance directives for cancer patients in Saudi Arabia

**DOI:** 10.1371/journal.pone.0213938

**Published:** 2019-04-12

**Authors:** Isamme N. AlFayyad, Mohamad A. Al-Tannir, Waleed A. AlEssa, Humariya M. Heena, Amani K. Abu-Shaheen

**Affiliations:** 1 Research Center, King Fahad Medical City, Riyadh, Saudi Arabia; 2 Comprehensive Cancer Center, King Fahad Medical City, Riyadh, Saudi Arabia; Boston University School of Medicine, UNITED STATES

## Abstract

This study aimed to investigate physicians’ and nurses’ knowledge and attitudes toward advance directives (ADs) for cancer patients, which empower patients to take decisions on end-of-life needs if they lose their capacity to make medical decisions. A cross-sectional study was conducted using convenience sampling. The outcomes were responses to the knowledge and attitude questions, and the main outcome variables were the total scores for knowledge and attitudes toward ADs. This study included 281 physicians and nurses (60.5%). Most physicians were men (95, 80.5%), whereas most nurses were women (147, 86.5%). The mean (standard deviation; SD) total knowledge score was 6.8 (4.0) for physicians and 9.1 (3.0) for nurses (p < 0.001). There was a significant difference in the total knowledge score between nurses and physicians, with an adjusted mean difference of 1.54 (95% confidence interval [CI]; 0.08–2.97). Other significant independent predictors of knowledge of ADs were female sex (1.60, 95% CI; 0.27–3.13) and education level (master’s versus bachelor’s: 1.26, 95% CI; 0.30–2.33 and Ph.D. versus bachelor’s: 2.22, 95% CI; 0.16–4.52). Nurses’ attitudes appeared to be significantly more positive than those of physicians, and the mean total attitude score (SD) was 19.5 for nurses (6.2) and 15.1 (8.1) for physicians (p < 0.001). The adjusted mean difference (95% CI) for nurses versus physicians was 3.71 (0.57–6.98). All participants showed a high level of knowledge of ADs; however, nurses showed considerably more positive attitudes than physicians.

## Introduction

Advanced care planning is a patient-centered mutual process that focuses on engaging them in treatment course. Healthcare providers assess patients’ values and preferences regarding their future care [1]. A significant part of these discussions is the streamlining and completion of advance directives (ADs), a legal document that empowers patients to make end-of-life (EOL) arrangements, particularly concerning their EOL care. This practice ensures that EOL needs are adequately met in the event of them losing their capacity to make medical decisions [2].

ADs aim to promote and protect the patient’s autonomy based on the conviction that patients who either choose a proxy decision maker, document their living will in advance, or both are more likely to receive essential care if they become incompetent. At least, the process assures noninterference with previously asserted healthcare plans [3,4].

Among patients with terminal malignancies, delirious status and cognitive impairments are highly prevalent [2]. EOL decisions are emotionally and intellectually challenging for patients and their healthcare providers [5]. Therefore, ADs must be discussed with a mentally competent patient who is not overwhelmed by the burden of the disease or other psychosocial and financial factors [6].

Several studies have reported the impact of ADs on improving the patient’s quality of life (QoL), enhancing patient-related outcomes, decreasing healthcare costs, decreasing in-hospital mortality rates, and optimizing the utilization of hospice service [6–10]. Furthermore, ADs promote the patient’s autonomy when stipulating their complete medical decisions, decrease the practice of physicians’ paternalism, and avoid any potential confrontation between the families and healthcare providers [11]. Conversely, the absence of ADs might perhaps lead to undesirable aggressive care when the patients have diminished decision-making capacity, compromise the patient’s QoL, and cause a pitiable bereavement for the caregivers as well [12,13]. However, despite the anticipated benefits, the rate of completion of ADs varies enormously [2,14–16].

Several factors affect the use of ADs, including patient and caregiver characteristics, patient’s perception of the AD objectives, and physicians’ and nurses’ limited knowledge of ADs [15,17–21]. Other factors comprise difficulties faced in articulating and interpreting ADs, reservations about their necessity, impact of tradition, hesitancy to converse openly about death, and preference of physicians or family members to make decisions on behalf of the patients when the latter becomes mentally incompetent [22]. Physicians’ attitudes toward the patient’s fear of becoming burdensome to families and having an undignified death were reported as essential factors influencing the completion of ADs [23].

Cultural dogmas, sociocultural beliefs, and values influence the meaning of death as well as the practice of EOL decision-making and use of ADs [19,24]. Although there is huge support for the use of ADs at EOL and several rules and regulations have been enacted worldwide, the Islamic world continues to lack literature on the debate surrounding AD legalization. In a single narrative article, Al-Jahdali (2012) described the Islamic perspective on ADs and reported that ADs are not as widely adopted by the Islamic community as by the West [25]. Hence, to systemically judge the need for and the value of ADs, we evaluated physicians’ and nurses’ knowledge and attitudes toward ADs.

## Materials and methods

### Study design and settings

A self-administered survey was developed for this descriptive cross-sectional study. Convenience sampling was used to recruit physicians and nurses providing care to cancer patients at three specialized medical areas (Cancer Center, National Neuroscience, and Intensive Care Unit) in a tertiary care medical city in Riyadh, Saudi Arabia.

### Participants and sample size

All physicians and nurses providing care to terminally ill cancer patients were offered the opportunity to participate in the study and complete the survey. In this study, 170 (response rate 70.8%) nurses and 111 (response rate 74%) physicians were recruited using nonprobability convenience sampling, and the survey was completed between August and December in 2017. convenience sampling was used to collect information from participants who were are easily accessible. Also, we assumed that the members of the target sample are homogeneous. Thus, there would be no difference in the research results obtained from a random sample.

### Survey instrument

The theory of planned behavior postulates that behavioral intentions and actual behavior are molded by the individual’s attitudes, normative beliefs, and perceived control over the behavior [26,27].

The development of the knowledge and attitude survey was steered by the theory of planned behavior and was designed on the basis of detailed literature reviews [25,28], author’s professional experiences in cancer care and clinical research, and the recommendations of two national palliative care experts.

The scale consisted of three main parts [S1 File]. The first part captured demographic data (age, gender, place of work, level of education, and total years of experience). The second part assesses the participants' knowledge using 12 questions related to the definition of ADs, types of ADs, living will, and the durable power of attorney. In addition, the questions assessed participants' knowledge of the onset of AD’s validity, itemizing of several clinical practices into the AD document (e.g., life-sustaining technology, cardiopulmonary resuscitation, and withholding nutrition and hydration), ideal timing of AD discussion, nomination of a person as a health care proxy, and the incorporation of that person in the discussion of ADs. The range of answers was “1 = yes” and “0 = no or I do not know.” Responses of “no” and “I do not know” were combined to score as they reflect the absence of knowledge. For the total knowledge scale, cutoffs (scores at 75th percentile) were considered as a satisfactory knowledge level (score: 8 out of 12).

The third part assesses the participants' attitudes using 27 questions, with the range of answers being “1 = yes” and “0 = no or I do not know.” The “no” and “I do not know” answers were combined as they reflect a lack of positive attitudes. The attitude questions were clustered into 4 subscales as follows: (i) planning of ADs (6 questions; cutoff score for positive attitudes is ≥4 out of 6), (ii) comfort and confidence in discussing ADs (8 questions; cutoff score for positive attitudes is ≥6 out of 8), (iii) application of ADs (9 questions; cutoff score for positive attitudes is ≥7 out of 9), and (iv) challenges of ADs (4 questions; cutoff score for positive attitudes is ≥3 out of 4). For the attitudes total scale and subscales, cutoffs (scores at 75th percentile) were considered as positive attitudes.

### Statistical analyses

Initially, the data were stored in SPSS version 21.0 (IBM Corporation, Armonk, NY, US) and converted into Stata version 15 for further analysis. Before the main analysis, the survey was piloted to assess the reliability and validity, besides identifying any difficulties or ambiguities. Internal consistencies in the knowledge (12 items) and attitude scales (27 items) were assessed using Cronbach’s alpha.

The participants’ responses to the questions were converted into binary variables (yes = 1 and “no” or “I do not know” = 0), and the total score for knowledge and attitudes toward ADs was computed. Correlation between the total scores for knowledge and attitudes was examined using Spearman’s correlation coefficient.

For the total scale and subscales of attitudes, cutoffs (scores at 75^th^ percentile) were considered as positive attitudes. Participants’ characteristics as well as knowledge and attitudes toward ADs were summarized separately for physicians and nurses using descriptive statistics, such as frequency, percentage, mean, and standard deviation (SD). To assess the differences between physicians’ and nurses’ responses, chi-square tests were used. Initially, the mean total scores for knowledge and attitudes were compared between physicians and nurses using *t*-tests and Mann–Whitney test. Receiver operating characteristics (ROC) curve analysis was used to examine whether the knowledge and attitudes scores discriminate between the professional groups (nurse versus physician). In this analysis, the area under the ROC curve (AUC) is calculated to show, how well the knowledge can distinguish between two groups of profession. As the knowledge and attitude scores were not normally distributed, nonparametric regression analysis with bootstrap method was employed to examine the adjusted differences between the physicians and nurses and to identify further predictors of knowledge and attitude. In nonparametric regression, the functional form between the outcome and the covariates was not specified. In regression analysis, the total scores for knowledge and attitudes were considered as dependent variables, and the covariates assessed were profession (nurse versus physician), sex, age, and education level. From the regression analysis, coefficients (adjusted mean differences of the scores) with 95% bootstrapped confidence intervals (CIs) were reported.

### Ethical consideration

The study was approved by the Institutional Review Board (IRB log #:16–369) of King Fahad Medical City. The participants were provided with a cover letter describing the study objectives. Anonymity was maintained and informed consent was implied by the completion of the survey.

## Results

### Reliability and validity of the knowledge and attitude scales

Cronbach’s alpha revealed that the knowledge scale reached an acceptable reliability of α = 0.88. Internal consistencies in the total attitude scale and four subscales were computed, and the following results were obtained: total attitude scale (27 questions), α = 0.93; AD planning (6 questions), α = 0.89; comfort and confidence (8 questions), α = 0.67; application of ADs (9 questions), α = 0.79; and challenges of ADs (4 questions), α = 0.71. The internal consistency value of the total attitude scale suggests that the questions fitted well to measure the intended attitudes.

### Participants’ characteristics

A total of 111 physicians and 170 (60.5%) nurses participated in the study. Mean (SD) age of the physicians and nurses was 33.4 (7.6) and 33.9 (6.6) years, respectively. Most physicians were men (95, 80.5%), while most nurses were women (147, 86.5%). Eighty-five (77.3%) physicians and 81 (48.2%) nurses had <5 years of experience. Nearly two-thirds of the nurses, that is, 112 (67.9%) individuals and 66 physicians (60%) indicated receiving specialized education on ADs. The majority of physicians (69, 63.3%) and nurses (148, 93.1%) were bachelor’s degree holders. A statistically significant difference was found between the physicians and nurses in terms of sex, education level, and years of experience (p < 0.001; Table 1).

**Table 1:** Distribution of the study participants.

Demographics	Physician(n = 111)	Nurse(n = 170)	*p* value
**Age**	33.37 ± 7.57	33.90 ± 6.56	0.569
**Female sex**	16 (14.4%)	147 (86.5%)	<0.001*
**Education level**			
Bachelor’s	69 (63.3%)	148 (93.1%)	
Master’s	13 (11.9%)	11 (6.9%)	<0.001*
PhD	27 (24.8%)	0	
**Years of Experience**			
<5 years	85 (77.3%)	81 (48.2%)	
6–10 years	12 (10.9%)	49 (29.2%)	<0.001*
>10 years	13 (11.8%)	38 (22.6%)	
**Place of work**			
Cancer center	45 (45.5%)	66 (40.7%)	
Intensive care units	26 (26.3%)	54 (33.3%)	0.484
Neuroscience department	28 (28.3%)	42 (25.9%)	

*Significance at p < 0.05

### Knowledge of ADs

#### Physicians

As shown in Table 2, 72 (64.9%), 65 (58.6%), and 68 (61.8%) physicians provided the correct definitions for ADs, living will, and durable power of attorney, respectively. Fifty-one physicians (45.9%) reported that the appropriate time to discuss ADs is when the patient is terminally or seriously ill. Table 2 also reveals physicians’ knowledge regarding the items that can be incorporated into ADs. The responses that indicated poor knowledge (“no” or “I do not know”) fell into 5 main categories: 49 (44.1%) for life-sustaining technology, 48 (43.2%) for cardiopulmonary resuscitation, 70 (63.1%) for withholding nutrition and hydration, 35 (35.1%) for healthcare proxy, and 44 (40%) for the place of terminal care and death.

**Table 2:** Participants’ responses to the knowledge of AD questions by profession.

Knowledge of AD questions	Physicians (111)	Nurses (170)	*p* value
	Yes, n (%)		
Q1: Definition of ADs	72 (64.9)	140 (82.4)	<0.001*
Q2: Types of ADs	60 (55.1)	133 (79.6)	<0.001*
Q3: Definition of living will	65 (58.6)	145 (85.3)	<0.001*
Q4: Definition of durable power of attorney	68 (61.8)	131 (78.0)	0.003*
Q5: Onset of AD validity	62 (55.9)	130 (76.9)	<0.001*
Q6: Itemizing of life-sustaining technology into AD document	62 (55.9)	135 (79.4)	<0.001*
Q7: Itemizing of cardiopulmonary resuscitation into AD document	63 (56.8)	135 (79.4)	<0.001*
Q8: Itemizing of withholding nutrition and hydration into AD document	41 (36.9)	120 (70.6)	<0.001*
Q9: Itemizing of place of terminal care and death into AD document	66 (60)	133 (78.2)	0.001*
Q10: Ideal timing of discussing ADs	60 (54.1)	74 (43.8)	0.093
Q11: Nomination of a principal person as a healthcare proxy	72 (64.9)	125 (74.0)	0.103*
Q12: Incorporation of the healthcare proxy in the discussion of ADs	67 (62.6)	143 (86.1)	<0.001*
**Mean total knowledge score (SD)**	**6.8 (4.0)**	**9.1 (3.0)**	<0.001*

*Significance at p < 0.05

#### Nurses

The majority of nurses were able to correctly articulate the definitions of ADs (140, 82.4%), living will (145, 85.3%), and durable power of attorney (131, 78%). Slightly more than three-quarters said that ADs become effective when the patient is mentally incapable. Moreover, 95 (56.2%) participants reported that the most appropriate time to discuss ADs is when the patient is terminally or seriously ill (Table 2).

Table 2 also displays nurses’ responses regarding the items that can be incorporated into ADs according to the patient’s preferences and decision. The responses that suggested poor knowledge (“no” or “I do not know”) fell into 5 main categories: 50 (29.4%) for withholding nutrition and hydration, 35 (20.6%) for life-sustaining technology, 35 (20.6%) for cardiopulmonary resuscitation, 44 (26%) for healthcare proxy, and 37 (21.8%) for the place of terminal care and death.

### Difference in the knowledge of ADs between nurses and physicians

**Fig 1:**
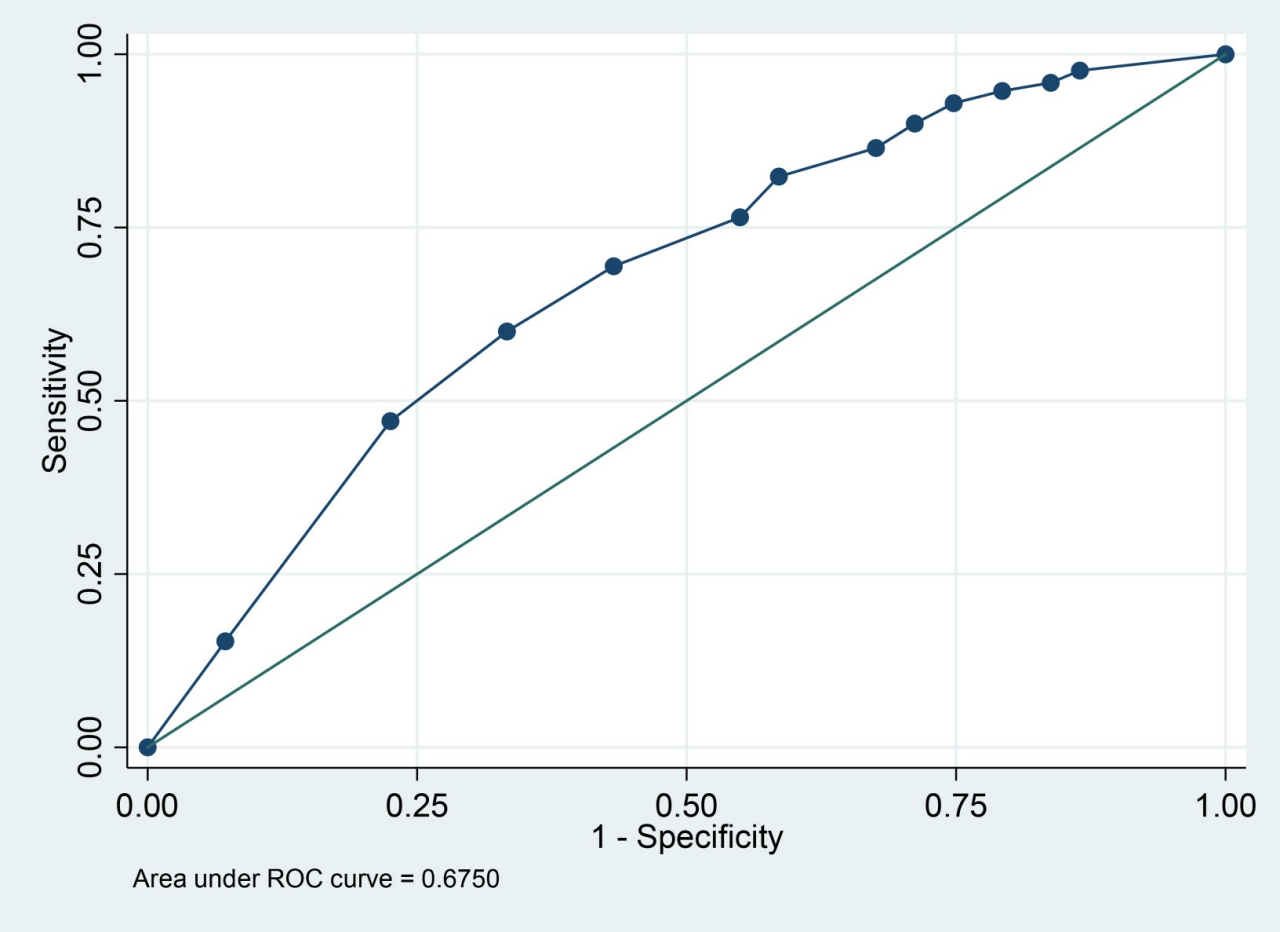
ROC curve analysis for the relationship between total knowledge score and profession (nurse versus physician).

A significant difference was observed in all the knowledge questions between the nurses and physicians, except for the questions related to “the most appropriate time for discussing AD” and “nomination of a principal person as a healthcare proxy” (*p* = 0.093 and 0.103, respectively; Table 2). The mean (SD) total knowledge score was 6.8 (4.0) for physicians and 9.1 (3.0) for nurses (p < 0.001). There was a significant difference in the total knowledge score between nurses and physicians, with an adjusted mean difference of 1.54 (95% CI; 0.08–2.97). Other significant independent predictors of the knowledge of ADs were female sex (1.60, 95% CI; 0.27–3.13) and education level (master’s versus bachelor’s: 1.26, 95% CI; 0.30–2.33 and Ph.D. versus bachelor’s: 2.22, 95% CI; 0.16–4.52; Table 3). Moreover, the sensitivity is plotted against (1-specificity) of the knowledge total score at various cut-off values. The area under a ROC curve (AUC) is a value between 0.5 and 1, which quantifies the overall ability of the knowledge score to discriminate between nurses and physicians. In this analysis AUC = 0.675 shows that nurses have higher knowledge score than physician. If the knowledge score would not decimate between nurses and physicians, the AUC would be 0.5. If the knowledge score would perfectly predict either of the professions, then the value of AUC would be 1.00. (Fig 1).

**Table 3:** Predictors of the knowledge of ADs.

Variables	Unadjusted effect estimates			Mutually adjusted effect estimates			
	β	Bootstrapped: (95% CI)	*p* values	β	Bootstrapped: (95% CI)		*p* values
Nurses vs. physicians	2.02	(1.20, 2.78)	<0.001	1.54	(0.08, 2.97)		**0.040**
Female vs male	2.18	(1.38, 2.90)	<0.001	1.60	(0.27, 3.13)		**0.023**
Education level							
Master’s	0.01	(−0.11, 0.12)	0.863	1.26	(0.30, 2.33)		**0.014**
PhD	0.01	(−0.14, 0.13)	0.979	2.22	(0.16, 4.52)		**0.050**
Age	0.08	(0.02, 0.13)	0.011	0.06	(−0.01, 0.14)		0.143

### Differences in attitudes toward ADs between physicians and nurses

Table 4 provides the results for the attitude toward ADs. Overall, physicians and nurses showed positive attitudes toward ADs. However, nurses’ attitudes appeared to be significantly more positive than those of physicians. The mean total attitude score (SD) was 19.5 (6.2) for nurses and 15.1 (8.1) for physicians (p < 0.001). Similar results were observed for the attitude subscales, with nurses performing better than physicians (Table 4). For example, in the case of AD planning, the subscale mean score (SD) for physicians was 3.2 (1.9), whereas that for nurses was 4.8 (1.6) (p < 0.001). Negative attitudes were more likely in physicians than in nurses when they were asked about the necessity of discussing ADs with every patient, irrespective of the diagnosis (36, 32.7% versus 120, 71.9%, p < 0.001), and with patients having life-threatening diseases who were willing to talk about their wishes for EOL care (66, 60.0% versus 135, 80.4%, p < 0.001). Both physicians and nurses appeared to have negative attitudes regarding the discussion of ADs as they diminish a sense of hope in patients. Moreover, positive attitude was more prevalent among physicians than nurses in their confidence of breaking the bad news to cancer patients (57, 51.8% versus 61, 36.5%, p = 0.012).

**Table 4:** Physicians and nurses’ attitudes toward ADs.

Subscale		Survey question	Physicians (N = 111)	Nurses (N = 170)	*p* value
			Yes, n (%)		
**Attitudes about AD planning** (Cronbach’s α: 0.79)	*Q1*	Discussion of ADs with every patient irrespective of the diagnosis	36 (32.7)	120 (71.9)	<0.001*
	*Q2*	Discussion of ADs with patients diagnosed with life-threatening diseases	66 (60.0)	135 (80.4)	<0.001*
	*Q7*	Discussion of ADs improves patients’ and families’ satisfaction with EOL care.	74 (67.3)	136 (81.9)	0.005*
	*Q9*	Discussion of ADs is the physician’s responsibility.	60 (55.1)	137 (81.6)	<0.001*
	*Q11*	Patients’ willingness to know their diagnosis, prognosis, and care options	68 (62.4)	147 (88.6)	<0.001*
	*Q12*	Patients’ willingness to communicate their wishes for EOL care	50 (45.9)	128 (76.9)	<0.001*
		**Mean total subscale score (SD)**	**3.2 (1.9)**	**4.8 (1.6)**	<0.001*
**Comfort and confidence** (Cronbach’s α: 0.67)	*Q3*	ADs decrease EOL care decisional catastrophe.	66 (60.6)	120 (71.4)	0.060
	*Q4*	Confidence in the treatment choices if directed by ADs	69 (63.3)	126 (76.4)	0.019*
	*Q5*	Less worry about legal consequences of limiting treatment if directed by ADs	74 (67.3)	127 (76.1)	0.109
	*Q6*	Discussion of ADs demolishes patients’ sense of hope.	39 (35.5)	59 (35.3)	0.983
	*Q13*	It feels easy when discussing matters related to EOL with patients and their families.	32 (29.1)	85 (50.9)	<0.001*
	*Q14*	Discussion of ADs produces confrontational relationship with the patient.	43 (39.1)	113 (67.7)	<0.001*
	*Q16*	It feels easy when discussing ADs with patients with progressive diseases.	30 (27.3)	100 (60.2)	<0.001*
	*Q17*	Confidence in breaking “bad news.”	57 (51.8)	61 (36.5)	0.012*
		**Mean total subscale score (SD)**	**3.7 (2.2)**	**4.7 (2.0)**	<0.001*
**Application of ADs** (Cronbach’s α: 0.89)	*Q8*	ADs decrease the likelihood of futile/unnecessary EOL care.	75 (68.8)	129 (77.3)	0.119
	*Q10*	Use of ADs is consistent with patient-centered care values in your health care institution.	66 (60.5)	136 (81.4)	<0.001*
	*Q18*	ADs decrease the cost of unnecessary treatment/care.	74 (67.9)	119 (72.1)	0.453
	*Q19*	ADs are useful in your institution	72 (66.7)	139 (83.2)	0.001*
	*Q20*	Your administration/colleagues would support the use of ADs.	65 (59.1)	124 (74.3)	0.008*
	*Q24*	ADs can be used in your institution if legalized.	66 (60.5)	133 (80.1)	<0.001*
	*Q25*	ADs positively affect the cost of total care and save medical expenditures in the long term.	78 (70.9)	135 (80.8)	0.055
	*Q26*	ADs improve and facilitate the discharge plan process.	79 (71.8)	137 (82.0)	0.045*
	*Q27*	Recommending your health care institution to adopt the use of ADs	78 (70.9)	143 (86.1)	0.002*
		**Mean total subscale score (SD)**	**5.9 (3.4)**	**7.1 (2.5)**	0.006*
**Challenges of ADs** (Cronbach’s α: 0.71)	*Q15*	A potential problem of ADs is that patients’ families could change their minds about treatment when the patient becomes terminally ill.	55 (50.0)	129 (78.2)	<0.001*
	*Q21*	ADs may be a relief for families in some circumstances.	73 (66.4)	135 (81.8)	0.003*
	*Q22*	ADs might be culturally accepted and established.	50 (45.5)	128 (77.6)	<0.001*
	*Q23*	ADs do not interfere with Islamic regulations.	63 (57.3)	94 (56.6)	0.916
		**Mean total subscale score (SD)**	**2.2 (1.5)**	**2.9 (1.2)**	<0.001*
**Mean total attitude score (SD**; Cronbach’s α: 0.93)			15.1 (8.1)	19.5 (6.2)	<0.001*

*Significance at p < 0.05

The adjusted mean difference (95% CI) for nurses versus physicians was 3.71 (0.57–6.98; Table 5). Participants’ age was also found to be a significant predictor of the total attitude score; higher score indicated advanced age (p < 0.001).

**Table 5:** Predictors of the participant’s attitudes toward ADs.

Variables	Unadjusted effect estimates			Mutually adjusted effect estimates			
	β	Bootstrapped: (95% CI)	*p* values	β	Bootstrapped: (95% CI)		*p* values
**Nurses vs. Physicians**	3.97	(2.45, 5.47)	<0.001	3.71	(0.57, 6.98)		0.026
**Female vs Male**	3.39	(2.05, 4.76)	<0.001	1.52	(−1.32, 4.78)		0.337
**Years of experience**	-	-	-				
*6–10 vs <5*	0.77	(0.19, 1.39)	0.012	-0.21	(−2.19, 1.29)		0.807
*>10 vs <5*	0.66	(0.00, 1.31)	0.043	-0.48	(−4.00, 2.43)		0.767
**Age**	0.23	(0.12, 0.36)	<0.001	0.25	(0.12, 0.41)		0.001
**Education level**							
*Master’s*	0.04	(−0.19, 0.29)	0.726	0.23	(0.12, 0.36)		<0.001
*PhD*	0.06	(−0.20, 0.30)	0.646	0.25	(0.12, 0.41)		0.001

**Fig 2:**
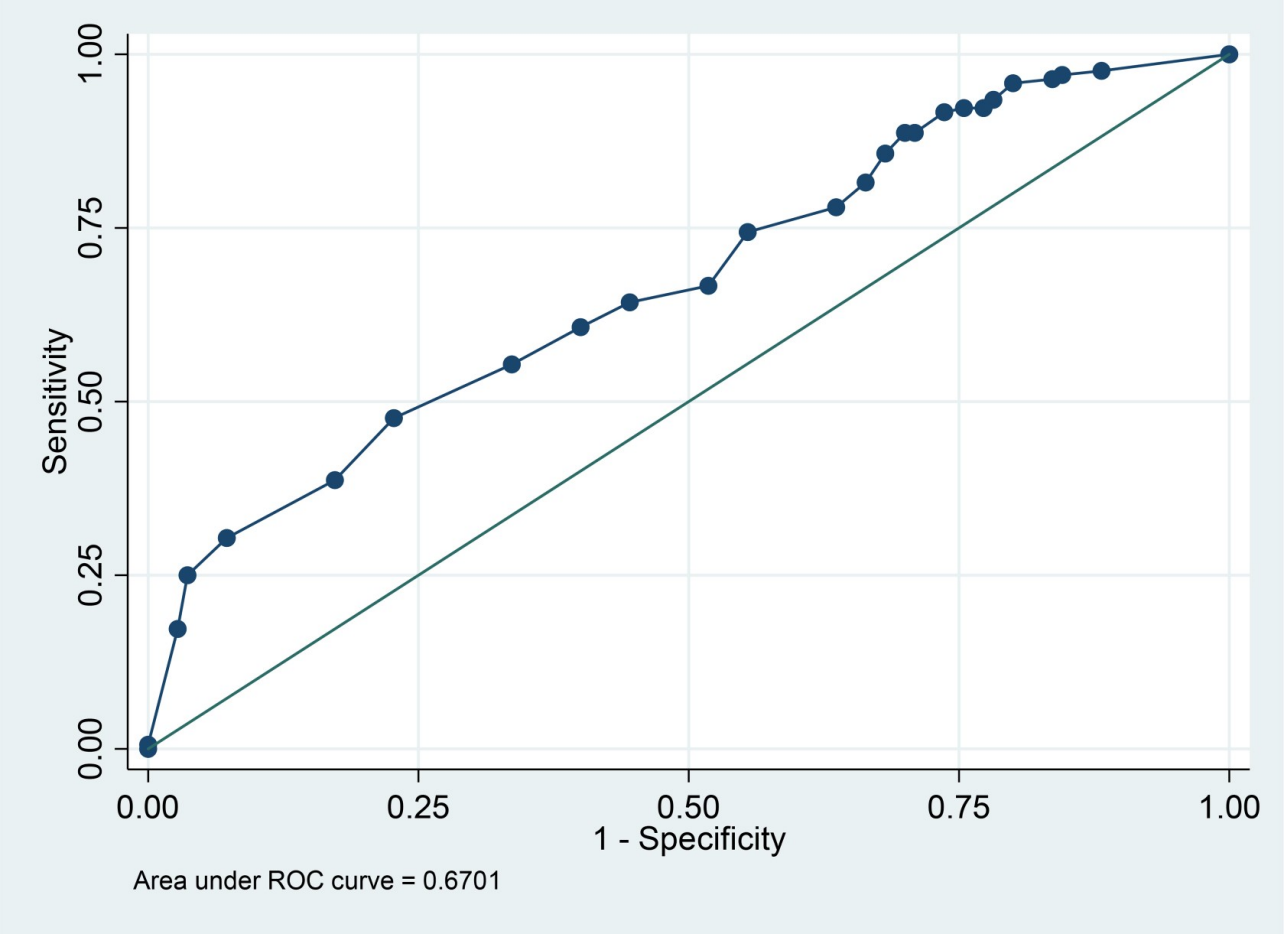
ROC curve analysis for the relationship between total attitude score and profession (nurse versus physician).

The total attitude scores and subscale scores correlated well with the total knowledge scores (p < 0.001). Spearman’s correlation coefficient for total scores was 0.576, and higher attitude scores predicted nurses (Table 6). The sensitivity is plotted against (1-specificity) of the attitude total score at various cut-off values. In this analysis AUC = 0.670 shows that nurses have higher attitude score than physician. If the attitude score would not decimate between nurses and physicians, the AUC would be 0.5. (Fig 2).

**Table 6:** Correlation between the total scores for knowledge and attitudes.

	Spearman’s correlation coefficient
Attitudes about AD planning	0.495*
Comfort and confidence	0.496*
Application of ADs	0.554*
Challenges of ADs	0.499*
Total attitude scale	0.576*

*Significance at p < 0.001

## Discussion

The results of this study aided in validating a new scale for the assessment of physicians’ and nurses’ knowledge and attitudes toward ADs. Overall, nurses were more knowledgeable about ADs and had a significantly more positive attitude than physicians. The multivariable analysis revealed that the total score for knowledge of ADs was significantly associated with the female sex and education level; higher education (master’s and Ph.D.) predicted elevated scores.

ADs empower cancer patients and their relatives to make informed decisions about EOL care. Efficiently providing ADs allows the healthcare institution to decrease the extra expenses that could be diverted to other healthcare priorities. Nevertheless, we hypothesized that the inscribed ADs could be principally authoritative in guiding the treatment of cancer patients.

Concerning the knowledge of ADs, nurses fared better in our study, similar to the cited data among US nurses [29–31]. Furthermore, the good knowledge may be attributed to the provision of AD education programs and in-service professional development in cancer nursing training. On the other hand, physicians lacked knowledge in the concept of ADs. We could attribute this observation to the absence of AD training, governing guidelines, unfamiliarity with the concept *per se*, or infrequent encounter with ADs. These findings are consistent with those derived from similar studies [32,33]. Physicians, nurses, and other caregivers should be adequately trained for proficiency in delivering ADs. We suggest that both physicians and nurses should complete AD training uniformly during their care and have access to AD registers.

There is variation in the provision of ADs for cancer care worldwide, and this study investigated a couple of variables that partly explain this phenomenon. The important one is the disparity between physicians’ and nurses’ knowledge of ADs and their attitudes toward them. Moreover, cultural dogmas, sociocultural beliefs, and values influence the meaning of death and dying, as well as the practice of EOL decision-making and use of ADs [19,24]. In addition, we argue that respect for the sanctity of life is a crucial value in Islamic societies, and this might have influenced physicians’ knowledge and attitudes toward ADs. The involvement of political, cultural, and religious leaders might also be required to design an efficient caregiving system and provide confidence to the professionals for delivering ADs and EOL care.

Healthcare system can dispel the myth that ADs are associated with imminent death [34], but instead enable more control over future care [35]. ADs offer patients the opportunity to manage their medical care should they ever lose their decision-making capacity. Furthermore, patients with ADs had better quality of care with reduced requirement of resuscitation and ventilation. Besides, they exerted control over the place of death and experienced the best utilization of the hospicecare; further, their caregivers also had superior QoL [2].

The strengths of this study include the large sample size representing the diverse care areas and the use of a culturally sensitive, valid, and reliable questionnaire. Our findings were limited by several factors including the use of convenience sampling and the single site of study. In addition, we did not investigate patient knowledge as a part of this research.

## Conclusion

A high level of knowledge of ADs was found among all participants; however, there was a significant disparity between nurses and physicians, with the former having considerably more positive attitudes than the latter. This may partly explain the variations in cancer care ADs in Saudi Arabia. Nonetheless, further investigations are required on this topic. This study describes a challenging and interesting subject that warrants further systematic work and opens a pertinent dialogue in the field of cancer care.

## Supporting information

S1 File KAAD scale: Knowledge and attitudes towards advance directives. (DOCX)

S2 File ADs data set. (SAV)

## References

[1] Sharma UM, Schroeder JE, Al-Hamadani M, Mathiason MA, Meyer CM, Frisby KA, et al An exploratory study of the use of advance directives by US oncologists. WMJ. 2013;112: 158–161. 24734404

[2] Dow LA, Matsuyama RK, Ramakrishnan V, Kuhn L, Lamont EB, Lyckholm L, et al Paradoxes in advance care planning: the complex relationship of oncology patients, their physicians, and advance medical directives. J Clin Oncol. 2010;28: 299–304. 10.1200/JCO.2009.24.6397 19933909 PMC2815718

[3] Yun YH, Han KH, Park S, Park BW, Cho CH, Kim S, et al Attitudes of cancer patients, family caregivers, oncologists and members of the general public toward critical interventions at the end of life of terminally ill patients. CMAJ. 2011; 183: E673–E679. 10.1503/cmaj.110020 21624907 PMC3134758

[4] Johnson S, Butow P, Kerridge I, Tattersall M. Advance care planning for cancer patients: a systematic review of perceptions and experiences of patients, families, and healthcare providers. Psychooncology. 2016;25: 362–386. 10.1002/pon.3926 26387480

[5] Pfirstinger J, Bleyer B, Blum C, Rechenmacher M, Wiese CH, Gruber H. Determinants of completion of advance directives: a cross-sectional comparison of 649 outpatients from private practices versus 2158 outpatients from a university clinic. BMJ Open. 2017;7: e015708 10.1136/bmjopen-2016-015708 29273648 PMC5778305

[6] Butler J, Binney Z, Kalogeropoulos A, Owen M, Clevenger C, Gunter D, et al Advance directives among hospitalized patients with heart failure. JACC Heart Fail. 2015;3: 112–121. 10.1016/j.jchf.2014.07.016 25543976

[7] Garrido MM, Balboni TA, Maciejewski PK, Bao Y, Prigerson HG. Quality of life and cost of care at the end of life: the role of advance directives. J Pain Symptom Manage. 2015;49: 828–835. 10.1016/j.jpainsymman.2014.09.015 25498855 PMC4441858

[8] Nicholas LH, Langa KM, Iwashyna TJ, Weir DR. Regional variation in the association between advance directives and end-of-life medicare expenditures. JAMA. 2011;306: 1447–1453. 10.1001/jama.2011.1410 21972306 PMC3332047

[9] Fonk J, Davidoff D, Lutzow T, Chesley N, Mathiowetz N. The effect of advance directives on end-of-life cost experience. J Health Care Poor Underserved. 2012;23: 1137–1156. 10.1353/hpu.2012.0098 24212165

[10] Brinkman-Stoppelenburg A, Rietjens JA, van der Heide A. The effects of advance care planning on end-of-life care: a systematic review. Palliat Med. 2014;28: 1000–1025. 10.1177/0269216314526272 24651708

[11] Dumitraș S, Enache M, Parvu A, Moisa SM, Iov C, Ioan B. Advance directive from the romanian social cultural perspective. Postmodern Openings. 2013;4: 87–102.

[12] Harrington SE, Smith TJ. The role of chemotherapy at the end of life: “when is enough, enough?”. JAMA. 2008;299: 2667–2678. 10.1001/jama.299.22.2667 18544726 PMC3099412

[13] Wright AA, Zhang B, Ray A, Mack JW, Trice E, Balboni T, et al Associations between end-of-life discussions, patient mental health, medical care near death, and caregiver bereavement adjustment. JAMA. 2008;300: 1665–1673. 10.1001/jama.300.14.1665 18840840 PMC2853806

[14] Sahm S, Will R, Hommel G. What are cancer patients’ preferences about treatment at the end of life, and who should start talking about it? A comparison with healthy people and medical staff. Support Care Cancer. 2005;13: 206–214. 10.1007/s00520-004-0725-z 15657689

[15] Silveira MJ, Kim SY, Langa KM. Advance directives and outcomes of surrogate decision making before death. N Engl J Med. 2010;362: 1211–1218. 10.1056/NEJMsa0907901 20357283 PMC2880881

[16] Antolín A, Sánchez M, Miró Ò. Temporal trend in understanding of and attitudes to advance directives in patients with chronic diseases. Gac Sanit. 2011;25: 412–418. 10.1016/j.gaceta.2011.03.009 21737187

[17] Ho GW, Skaggs L, Yenokyan G, Kellogg A, Johnson JA, Lee MC, et al Patient and caregiver characteristics related to completion of advance directives in terminally ill patients. Palliat Support Care. 2017;15: 12–19. 10.1017/S147895151600016X 27237410 PMC8976443

[18] Del Pozo Puente K, Hidalgo JL, Herráez MJ, Bravo BN, Rodríguez JO, Guillén VG. Study of the factors influencing the preparation of advance directives. Arch Gerontol Geriatr. 2014;58: 20–24. 10.1016/j.archger.2013.07.009 23993265

[19] Andres-Pretel F, Navarro BB, Párraga IM, de la Torre García MA, Jiménez Del Val MD, López-Torres Hidalgo J. Seniors’ knowledge of and attitudes to advance directive documents. Gac Sanit. 2012;26: 570–573. 10.1016/j.gaceta.2011.12.007 22444520

[20] Navarro BB, Sánchez MG, Andrés FP, Juárez IC, Cerdá RD, Párraga IM, et al Living will declarations: Qualitative study of the elderly and primary care general practitioners. Aten Primaria. 2011;43: 11–17. 10.1016/j.aprim.2010.01.012 20304533 PMC7024500

[21] Tobler D, Greutmann M, Colman JM, Greutmann-Yantiri M, Librach SL, Kovacs AH. Knowledge of and preference for advance care planning by adults with congenital heart disease. Am J Cardiol. 2012;109: 1797–1800. 10.1016/j.amjcard.2012.02.027 22459306

[22] Hinders D. Advance directives: limitations to completion. Am J Hosp Palliat Care. 2012;29: 286–289. 10.1177/1049909111419293 21868429

[23] Alano GJ, Pekmezaris R, Tai JY, Hussain MJ, Jeune J, Louis B, et al Factors influencing older adults to complete advance directives. Palliat Support Care. 2010;8: 267–275. 10.1017/S1478951510000064 20875170

[24] Johnson KS, Kuchibhatla M, Tulsky JA. What explains racial differences in the use of advance directives and attitudes toward hospice care? J Am Geriatr Soc. 2008;56: 1953–1958. 10.1111/j.1532-5415.2008.01919.x 18771455 PMC2631440

[25] Al-Jahdali H, Baharoon S, Al Sayyari A, Al-Ahmad G. Advance medical directives: a proposed new approach and terminology from an Islamic perspective. Med Health Care Philos. 2013;16: 163–169. 24571002 10.1007/s11019-012-9382-z

[26] Rivis A, Sheeran P. Descriptive norms as an additional predictor in the theory of planned behavior: A meta-analysis. Curr Psychol. 2017;22: 49–68.

[27] Detering KM, Hancock AD, Reade MC, Silvester W. The impact of advance care planning on end of life care in elderly patients: randomised controlled trial. BMJ. 2010;340: c1345 10.1136/bmj.c1345 20332506 PMC2844949

[28] Zhou G, Stoltzfus JC, Houldin AD, Parks SM, Swan BA. Knowledge, attitudes, and practice behaviors of oncology advanced practice nurses regarding advanced care planning for patients with cancer. Oncol Nurs Forum. 2010;37: E400–E410. 10.1188/10.ONF.E400-E410 21059573

[29] Coffey A, McCarthy G, Weathers E, Friedman MI, Gallo K, Ehrenfeld M, et al Nurses’ knowledge of advance directives and perceived confidence in end of life care: a cross sectional study in five countries. Int J Nurs Pract. 2016;22: 247–257. 10.1111/ijn.12417 26823112 PMC5066738

[30] Walerius T, Hill PD, Anderson MA. Nurses’ knowledge of advance directives, patient self-determination act, and Illinois advance directive law. Clin Nurse Spec. 2009;23: 316–320. 10.1097/NUR.0b013e3181be3273 19858904

[31] Putman-Casdorph H, Drenning C, Richards S, Messenger K. Advance directives: evaluation of nurses' knowledge, attitude, confidence, and experience. J Nurs Care Qual. 2009;24: 250–256. 10.1097/NCQ.0b013e318194fd69 19525766

[32] Aguilar-Sánchez JM, Cabañero-Martínez MJ, Puerta FF, Ladios-Martín M, Fernández-de-Maya J, Cabrero-García J. Knowledge and attitudes of health professionals towards advance directives. Gac Sanit. 2017. 10.1016/j.gaceta.2017.08.006 29110888

[33] Luk Y, Ngai C, Chau SS, Lam MY, Wong OW, Holm M. Clinicians’ experience with and attitudes toward discussing advance directives with terminally ill patients and their families in a Chinese community. J Palliat Med. 2015;18: 794–798. 10.1089/jpm.2015.0104 26186241

[34] Ganti AK, Lee SJ, Vose JM, Devetten MP, Bociek RG, Armitage JO, et al Outcomes after hematopoietic stem-cell transplantation for hematologic malignancies in patients with or without advance care planning. J Clin Oncol. 2007;25: 5643–5648. 10.1200/JCO.2007.11.1914 18065735

[35] Perkins HS. Controlling death: the false promise of advance directives. Ann Intern Med. 2007;147: 51–57. 17606961 10.7326/0003-4819-147-1-200707030-00008

