# Supplementary material for: Physicians and nurses’ knowledge and attitudes towards advance directives for cancer patients in Saudi Arabia
Source: PLoS One. 2019 Apr 12;14(4):e0213938. doi: 10.1371/journal.pone.0213938 (PMC6461283; doi:10.1371/journal.pone.0213938)
Supplement: S1 File — (DOCX) [file pone.0213938.s001.docx]

**K**nowledge and **A**ttitudes towards **A**dvance **D**irectives for cancer patients in Saudi Arabia

**(KAAD) Scale**

| - **Demographics data:** | | | | |
| --- | --- | --- | --- | --- |
| **Age** (years): | **Gender:**  Male Female | | | |
| **Years of Experience** (years)**:** | | | | |
| **Place of Specialty:** | | | | |
| **Practice specialty:**  Physician  Nurse | | | | |
|  | | | | |
| - **Health Care professionals Knowledge regarding Advance Directives:** | | | | |
| **Items** | | **Yes** | **No** | **I do not know** |
| 1. An advance directive is a legal document that informs the physician about patients’ wishes earlier about future health care if he/she becomes mentally incompetent. | |  |  |  |
| 1. The types of advance directives are the living will and the durable power of attorney for health care: | |  |  |  |
| 1. The living will is a document aims to govern specific future health care decisions merely when a patient becomes incapable to make decisions on their own. | |  |  |  |
| 1. A durable power of attorney for health is an official document in which patient designates a person to be his/her proxy to make all his/her health care decisions if he/she becomes incapable. | |  |  |  |
| 1. An advance directive will not influence the type or quality of patient care while he/she can express his/her decisions. It only becomes effective when he/she mentally cannot do so? | |  |  |  |
| 1. In the advance directives, the patient can decide whether or not to use life-sustaining machines, like a mechanical ventilator and dialysis. | |  |  |  |
| 1. In the advance directives, the patient can decide whether or not to have a CPR or DNR. | |  |  |  |
| 1. In the advance directives, the patient can decide whether or not to withhold nutrition and hydration. | |  |  |  |
| 1. In the advance directives, the patient can decide the place of terminal care and death. | |  |  |  |
| 1. The most appropriate time to discuss advanced directive is when the patient is terminally or seriously ill. | |  |  |  |
| 1. In an effective advanced directive communication, it is imperative to ask the patient to nominate a principal person as a health care proxy. | |  |  |  |
| 1. It is imperative to include the patient’s healthcare proxy in the discussion of advance directive | |  |  |  |
| - **Health Care professionals Attitudes toward advance directives:** | | | | |
| 1. The advanced directive has to be discussed with every patient irrespective of his/her diagnosis. | |  |  |  |
| 1. Discussion of the advanced directive is imperative to patients who are diagnosed with life-threatening diseases. | |  |  |  |
| 1. The advanced directive could lessen the end-of-life care decisional catastrophe. | |  |  |  |
| 1. In a catastrophic situation, you would have more confidence in the treatment choices if directed by an advance directive. | |  |  |  |
| 1. You would worry less about legal consequences of limiting treatment if you were following an advance directive. | |  |  |  |
| 1. Discussion of advanced directive could end patients’ sense of hope. | |  |  |  |
| 1. Discussion of advanced directive could improve patients’ and families’ satisfaction with end-of-life care. | |  |  |  |
| 1. Advanced directive reduces the use of futile/unnecessary care at the end of life. | |  |  |  |
| 1. Discussion of the advanced directive is the physician’s responsibility. | |  |  |  |
| 1. Practicing advanced directive could be consistent with patient-centered care standards in your health care institution. | |  |  |  |
| 1. Most of your patients are willing to know their diagnosis, prognosis, and care options. | |  |  |  |
| 1. Most patients with end stages diseases are willing to communicate their wishes for end-of-life care. | |  |  |  |
| 1. In your culture, it feels easy when discussing matters related to the end of life with patients and their families. | |  |  |  |
| 1. In your culture, discussion of an advance directive would produce a more confrontational relationship with the patient. | |  |  |  |
| 1. A prospective problem with advance directives is that patients’ families could change their minds about treatment when their patient becomes terminally ill. | |  |  |  |
| 1. In your culture, it feels easy when discussing advanced directive with patients with progressive diseases. | |  |  |  |
| 1. I feel confident in my ability to communicate “bad news.” | |  |  |  |
| 1. The advanced directive in long term reduces the cost of unnecessary treatment/care. | |  |  |  |
| 1. Advance directive document could be useful in your institution | |  |  |  |
| 1. Your administration/colleagues would support the practice of advanced directive. | |  |  |  |
| 1. The advance directive may be a relief for families in some circumstances. | |  |  |  |
| 1. The advance directive might be culturally accepted and established. | |  |  |  |
| 1. The advance directive does not interfere with the Islamic regulations. | |  |  |  |
| 1. The advance directive can be applied in your institution if legalized. | |  |  |  |
| 1. The advance directive in long term affects positively the cost of total care and save medical expenditures. | |  |  |  |
| 1. The advance directive can improve and facilitates the discharge plan process. | |  |  |  |
| 1. You would recommend your health care institution to adopt the practice of advance directive. | |  |  |  |
